# Dual versus triple therapy in treatment of hepatitis C virus (HCV)

**DOI:** 10.1007/s11845-022-03120-9

**Published:** 2022-08-30

**Authors:** Nevine Bishai, Walid el Nabawy, Mohamed El Fiki, Mohamed Ibrahim, Nouman El Garem

**Affiliations:** 1 Department of Internal Medicine, Faculty of Medicine, Cairo University Hospitals, Kasr El Aini, Cairo, Egypt; 2 Faculty of Medicine, Beni-Sueif University Hospitals, Beni Sueif, Egypt; 3 Faculty of Medicine, Cairo University Hospitals, Kasr El Aini, Cairo, Egypt

**Keywords:** Daclatasvir, Egypt, HCV, Interferon, Ribavirin, Sofosbuvir, SVR

## Abstract

**Background:**

The goal of HCV treatment is eradication of the virus to prevent complications associated with the disease and decrease all-cause mortality. This work compared sustained viral response (SVR) 12 weeks after end of treatment of chronic HCV patients with different treatment regimens, namely 4 regimens. Two hundred treatment naive chronic HCV patients were selected and divided into 4 equal groups as follows: group A received pegylated interferon (peg IFN) and ribavirin (RBV); group B received peg IFN, RBV, and sofosbuvir (SOF); group C received RBV and SOF; group D received SOF, daclatasvir (DCV), and RBV.

**Results:**

The sustained viral response after 12 months of treatment is 57.23%, 72.09%, 64.40%, and 96.42% of patients in groups A, B, C, and D, respectively. Hence, group D regimen showed the best results.

**Conclusion:**

SOF and DCV and RBV have the highest SVR12 and least side effects compared to other treatment regimens. Although group D patients initially had poor pretreatment investigations relative to other groups, they proved to have the highest tolerability to this regimen. Such findings hold promising line of treatment and better prognosis even for chronic HCV patients with poor liver condition.

## Introduction

Hepatitis C virus (HCV) is estimated to affect more than 200 million people, with an estimated HCV prevalence of 2.2% globally [1]. In Egypt, HCV prevalence is around 14.7%, not to mention that Egypt has the highest rate of spread 1 in 10 of the age groups 15 to 59 with a mortality rate of about 40,000/year [2]. Most patients infected with HCV remain chronically infected, with an increased risk of cirrhosis and hepatocellular carcinoma [3]. Eradication is confirmed by the measurement of sustained virologic response (SVR) defined as the absence of HCV RNA in blood 3 months after hepatitis C therapy is complete [4].

As our country reflects on the newest direct antiviral drugs that have been approved, more careful evaluation of the total cost of therapy and realistic expectations about the outcomes must be fully analyzed. In this study, we will be looking at different treatment regimens, including different treatment protocols, such as interferon, ribavirin, sofosbuvir, and daclatasvir.

## Materials and methods

### Patients

Between December 2015 and July 2021, patients were taken from the national virology center in BeniSeuif. Patients, eligible for enrollment, met the following criteria: naïve patients who have not received any treatment earlier, patients’ age ranged from 18 to 80 years. Compensated child A and B, patients were tested for FBS, albumin, TSH, CBC, liver functions, ANA, viral hepatitis, and alpha fetoptn. According to Omran et al. and Kouyoumjian et al. in [5], the majority of Egyptian patients infected around 92.5% are genotype 4. HCV patients who refused taking the antiviral treatment were set as a control group.

### Exclusion criteria

Hb < 10 mg/dl, clinical or biochemical signs of hepatic decompensation, decompensated child C, cardiac patients receiving amiodarone, hepatic encephalopathy or history of it, and hepatic or extrahepatic malignancy were all excluded. Patients were selected from the national virology center in Beni Sueif. There was no financial fund for this study. Written informed consent was obtained from all patients and this study conformed to the ethical guidelines.

### Clinical and laboratory assessment

Hematological and biochemical tests were performed before starting (EVR), 4 weeks after starting treatment (EOR), and 12 weeks after treatment (SVR12). ECG, fundus examination for patients > 40 years, and upper endoscopy were also performed to exclude varices. Elastagrophy (ultrasound and fibro scan) was used to assess spleen and liver stiffness by Fib4 score prior to treatment. These parameters were measured by standard laboratory techniques at Central Laboratories, Benisuif University Hospitals.

### Measurement of HCV RNA

HCV RNA was measured by real-time PCR, Bosphore HCV kit from Anatolia Genworks, with a lower limit of quantification 15 IU/ml. Early virological response (EVR) when starting treatment; end of treatment response (EOR) 4 weeks after therapy and sustained viral response (SVR) 12 weeks after completion of therapy.

### Antiviral treatment

Patients were divided into four main groups: each receiving a different type of therapy. Group A and B receiving interferon needed more strict inclusion criteria such as follows: Hb > 13 g/dl for male, Hb > 12 g/dl for female, TLC > 400/mm^3^, plt > 100,000mm^3^, T.Bil < 12 mg/dl, albumin > 3.5 g/dl, no autoimmune, cardiac diseases, esophageal or gastric varices.

### Antiviral treatment (procedure)

Patients receiving interferon were given Pegsays 180. Patients receiving interferon and ribavirin were given in a weight-dependent dose, i.e., 1200 mg/day if body weight > 75 kg or 1000 mg/day if body weight < 75 kg. Patients who showed drop in Hb less than 10 g/dl were given a reduced dose of ribavirin by half and stopped if it dropped to < 8.5 g/dl. On the other hand, sofosbuvir was given in a dose of 400 mg single oral dose and declatasvir was given as 60 mg single oral dose. If patients developed drop in HB below 8.5 or GIT troubles in terms of nausea, vomiting, abdominal discomfort, splenomegaly and cirrhosis, anemia, hypersensitivity reaction, etc., treatment was to stop.

In this study, we examined 200 patients with chronic HCV infection, and they were divided into four equal groups:

- Group A: 50 patients the *dual therapy* with only pegylated interferon and ribavirin;
- Group B: 50 patients took the *triple therapy* regimen (pegylated interferon, ribavirin, and sofosbuvir);
- Group C: 50 patients took sofosbuvir and ribavirin alone (for patients not fit for interferon therapy);
- Group D: 50 patients took sofosbuvir plus daclatasvir and ribavirin.

### Statistical analysis

Data were expressed as a mean ± SD; statistical analysis was performed using Student’s *t*-test or chi-square test with Excel statistics program for window 7. *P* value of less than 0.05 was considered statistically significant.

## Results

### Patients’ characteristics

Clinical characteristics of patients in the present study are shown in Table 1–of the total of 200 patients, 50 patients were assigned in each group receiving different therapy.

**Table 1:** Patients’ characteristics in each group prior to treatment

Patients’ characteristics	Group A	Group B	Group C	Group D
Age (mean)	48.48 ± 7.32	49.86 ± 8.41	58.10 ± 6.76	57.78 ± 8.98
Gender (male %)	70	75	78	72
Splenomegaly(#)	4	3	7	4
Cirrhosis (#)	6	7	10	11

Table 1 shows that the mean age of patients in the first two groups, who received interferon in their regimen, is younger than the last two groups who received interferon free regimens. Group A and group B had a mean age of 48.8 and 49.86, respectively; on the other hand, group C and D had 58.1 and 57.57, respectively. However, the overall mean age for patients in the four groups was higher than 48. It is also worth noting that there is statistically significant male predominance among the groups. The percentage of male patients included outnumbered the percentage of females in all the groups. To illustrate, as can be depicted from Table 1, male compromised 70% of group A, 75% of group B, 78% of group C, and 72% of group D.

According to Table 1, the percentage of patients having cirrhosis and splenomegaly are mainly in the interferon free regimen therapy (group C and D), as these patients are more liable to interferon complications. Nevertheless, in spite of the relatively poor condition of the liver and spleen, these groups proved to respond better to treatment as will be mentioned later in Table 4.

### Associated comorbidities

**Fig. 1:**
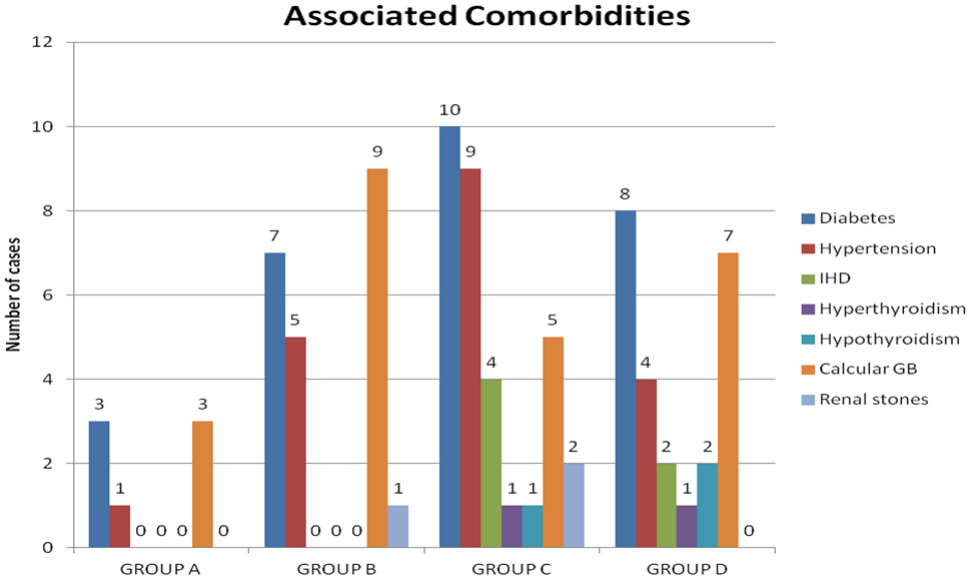
Number of patients with different associated comorbidities in each group

The bar chart in Fig. 1 shows the number of patients who had associated comorbidities. Common associated diseases are diabetes, hypertension, thyroid problems, renal stones, and calcular GB. Diabetes, hypertension, and calcular GB were associated with the four groups. Diabetes and hypertension were mostly prevalent in group C (10) and (9), respectively. Thyroid problems were only encountered with a low rate in group C and group D. Nevertheless, there was none suffering from thyroid or ischemic heart disease (IHD) in interferon taking group cause of its negative impact on thyroid [6] and cytotoxicity to heart [7], although calcular GB is associated with the four groups, it was mostly encountered with group B (9). It is worth noting that the most prevalent comorbidity is diabetes with groups C and D having the highest percentage among the four groups with 10 patients in group C and 8 patients in group D, versus 3 in group A and 7 in group B.

### Associated side effects during treatment

**Fig. 2:**
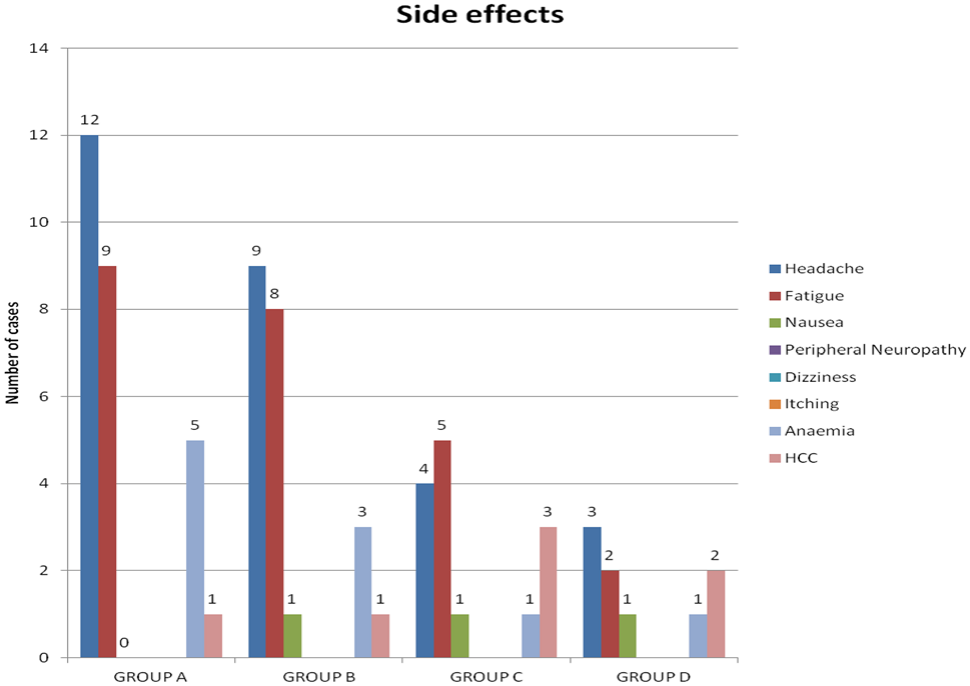
Number of patients in each group who developed different associated side effects during the course of study

The bar chart in Fig. 2 shows that the number of patients experiencing side effects is mainly in group A and B with headache and fatigue being the most commonly encountered. Group A patients developed the most common side effects, 12 patients having headache, 9 patients fatigue, and 5 patients developed anemia. Group D, on the other hand, developed the least side effects as compared to the first three groups. Although hepatocellular carcinoma was slightly higher in group C and D, this developed early in the course of treatment as the patients’ liver pretreatment condition was much worse than that of group A or B as shown in Tables 1 and 2. However, it was documented that five patients stopped treatment in group A because they developed anemia (< 7 g/dl) and four patients stopped treatment in group B (one could not tolerate treatment cause of severe nausea and vomiting and three developed anemia (< 7 g/dl)).

**Table 2:** Mean lab investigation (pretreatment)

	Group A	Group B	Group C	Group D
Bilirubin	0.89 ± 0.44	0.789 ± 0.377	0.98 ± 0.56	1.43 ± 2.97
AST	49.23 ± 25.12	48.74 ± 36.41	51.15 ± 33.99	58.03 ± 45.56
ALT	55.12 ± 30.11	64.18 ± 55.64	53.15 ± 35.92	58.83 ± 48.96
Hb	13.12 ± 2.10	15.11 ± 1.65	14.30 ± 1.50	14.36 ± 1.66
WBC	6.54 ± 1.73	6.95 ± 1.87	6.54 ± 2.36	6.37 ± 1.69
PLT	190.08 ± 40.15	189.05 ± 59.86	171.125 ± 74.21	183.34 ± 79.8
PCR	4,650*10^3^ ± 2.5*10^5^	4,955*10^3^ ± 4.2*10^5^	5,23010^3^ ± 4.8*10^5^	5,125010^3^ ± 1.5*10^5^

According to Tables 2 and 3, it is quite clear that liver markers and viral load were worse in the pretreatment interferon free regimen groups as there are less strict inclusion criteria and less post-treatment complications in comparison to those receiving interferon. However, after 12 weeks from treatment, liver functions improved with a pace much more than that in group A and B. For example, AST dropped from a mean of 58 to 27 in group D while from 49 to 48 only in group A. This may show how effective is the treatment regimen of the former. This improvement is not quite obvious in the blood picture parameters (Hb, WBCs, and PLTs) for pre- and post-treatment.

**Table 3:** The mean for lab results after 12 months of end of treatment (SVR12) for the four groups

Lab tests	Group A	Group B	Group C	Group D
Bilirubin	0.91 ± 0.21	0.64 ± 0.26	0.717 ± 0.50	0.874 ± 0.73
AST	48.33 ± 25.12	30.46 ± 21.53	30.17 ± 20.94	27.055 ± 12.53
ALT	54.13 ± 30.13	31.34 ± 27.69	28.10 ± 22.43	21.75 ± 13.85
Hb	13.112 ± 1.55	14.08 ± 2.02	13.87 ± 1.84	13.49 ± 15.62
WBC	6.31 ± 1.80	6.38 ± 2.32	6.748 ± 2.90	6.415 ± 1.96
PLT	192.11 ± 32.07	188.97 ± 60.61	193.58 ± 89.35	185.83 ± 73.8

### Percentage of responders vs non-responders of the four groups

Eradication is confirmed by the measurement of sustained virologic response (SVR). It is defined as the absence of HCV RNA in blood after hepatitis C therapy is complete. Sustained viral response 12 (SVR12 weeks after the end of treatment) was conducted for the four groups.

**Fig. 3:**
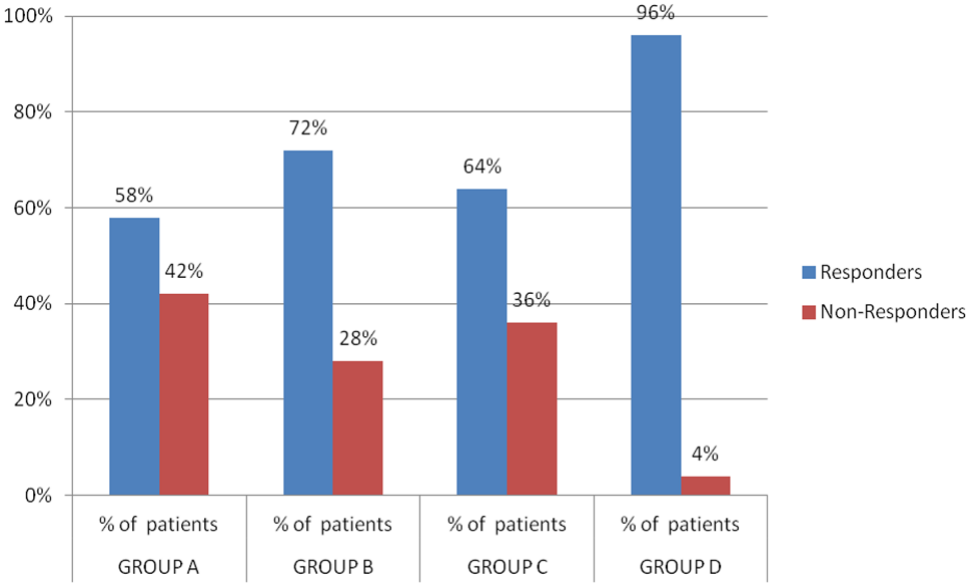
The percentage of patients who achieved SVR (responders) versus those who did not achieve SVR (non-responders)

The above results in Fig. 3 and Table 4 show that the highest SVR12 was achieved in group D with 96% followed by group B with 72%, then group C and group A with 64% and 58%, respectively. The reason for such variation in response rate will be discussed in details later.

**Table 4:** The percentage of the responders vs non-responders for the four groups

	Group A	Group B	Group C	Group D
Responders	58%	72%	64%	96%
Non-responders	42%	28%	36%	4%

### Safety

In the present comparative study, we tried to obtain information of the patients to discontinue treatment whenever needed. In patients of group A, five patients stopped as they developed anemia (Hb < 8.5 g/dl). In group B, four patients stopped treatment, one could not tolerate treatment developing severe nausea no vomiting, and three developed anemia (Hb < 8.5 g/dl).

## Discussion and conclusion

The current study is a longitudinal comparative study that aims at comparing the traditional dual regimen (group A) versus the triple regimen (group B), and with other novel treatment regimens groups C and D. Several parameters were used to assess the efficacy of the different regimens in 4 different stages of treatment, namely pretreatment, 1 month from the start of treatment, end of treatment, and SVR after 12 weeks. As Yoshida et al. in 2015 validated SVR12 as an efficient endpoint for the evaluation of regimens containing sofosbuvir and other direct-acting antiviral (DAA) medications, DAA are combinations of antivirals that work on different stages of the HCV life cycle, hence proved to be more effective than the old regimen such as ribavirin and interferon.

It was noticed in Fig. 1 that the mean age of patients in the first two groups, who received interferon in their regimen, is younger than the last two groups who received interferon free regimens. This could be attributed to the age restriction imposed on patients receiving IFN, as it was not eligible for patients above 60 years of age for fear of its complications.

It was also noted that there is a statistically significant male predominance among the 4 groups as per Fig. 2. This may be due to the fact that there is a worldwide male predominance not just in Egypt but in the whole world. In addition, this study was done in a rather conservative society as Beni Suef where most of those approaching the national virology center for screening and treatment were predominantly males.

Concerning the associated comorbidities among subjects in different groups, it was found that the most common comorbidities in the four groups were diabetes and hypertension. However, they are usually age-related, as their percentage increases with older age groups (group C and group D) as can be seen in Figs. 1 and 2. Other studies have also demonstrated that diabetes is associated with older age groups of chronically diseased HCV patients. This supports the idea that the occurrence of diabetes in HCV patients is progressive rather than abrupt [8]. This could be the reason why the number of diabetic patients in groups C and D was found to be higher than in groups A and B, as the mean age in groups C and D was more than that in groups A and B.

Associated side effects were more prominent in group A and B with headache and fatigue being the most commonly encountered as per Fig. 2. Yet, treatment has to be stopped for 5 patients and 3 patients in group A and B respectively who developed severe anemia. This is quite expected in these 2 groups receiving IFN as according to Bruno et al. [9] and Gramenzi et al. [10], peg IFN alone carries a number of side effects, poor intolerability, and increased toxicity.

In comparing the efficacy of different treatment regimes, SVR is the major indicator of efficacy as was stated by Yoshida (2015) and EASL (2016). SVR12 has been accepted as the endpoint of therapy both in the USA and in Europe, given that the “concordance” is more than 99% (Martinot-Peignous et al., 2010). It was stated that SVR corresponds to definitive cure of HCV infection in more than 99% of the cases [11].

When studying the SVR of the four groups as per Fig. 3 and Table4, the following conclusions were noticed respective to each group. To start with, the 50 treatment-naïve patients in group A who received IFN and ribavirin showed a SVR of 58%, which is comparable to that obtained by Kumar et al. in [12] where 121 patients receiving peg IFN α and ribavirin achieved SVR of 53%. Although different genotypes may show different response as Ward and Kugelmas [13], research showed SVR 80% with genotypes 2 and 3 and 45% with genotype 1, Kemp and Roberts [14] assured that such therapy had SVR from 45 to 56% across all genotypes.

Considering group B, the SVR rates in our study (72%) were lower than that obtained by either Elmashad et al. [15] (84%) or Dolatimehr et al. [16] (88%) or Kowdley et al. [17] (89%). This higher SVR12 could be due to the fact that the abovementioned studies included mainly genotype 1 patients; maybe if it included other genotypes, the results could have been different, as most of Egyptians are genotype 4 Omran et al. [19, 18] and Kouyoumjian et al. in [5]. Nevertheless, the results obtained in our study were found to have a SVR rate higher than that obtained in a study done by Aboud et al. in 2017 (54%) who evaluated the efficacy of triple therapy of sofosbuvir, ribavirin, and IFN in treatment of chronic HCV patients having fibrosis. This could be attributed to the fact that in Aboud study, all the patients chosen had high degree of fibrosis, while the patients in our study were mainly child A which could explain the higher SVR rate [19].

In comparing SVR of group C in our study (64%) to that in the POSITRON study (78%) and TARGET study (88.2%), the former was significantly lower. This may be due to the fact that the current study was conducted mainly on Egyptian patients who are mostly genotype 4 Kouyoumjian et al. [5], while the POSITRON study was conducted only on patients with genotype 2 or 3 [20]. While group C showed SVR12 64%, Doss et al. [18] who conducted his work on genotype 4 Egyptian patients showed 77%,this is mainly due to the fact that 20% of the former are cirrhotic as shown in Table 1 which in turn had a negative impact on SVR12. According to Doss, cirrhotic patients had lower rates of 63% than non-cirrhotic ones which is almost comparable to the presented results.

The highest SVR (96%) was witnessed with group D who took sofosbuvir plus daclatasvir; this was rather close to that achieved in a number of lab trials, for example, in phase IIA of LONESTAR open-label trial where the SVR was approximately 98%, 92%, and 89% for of genotype 1, 2, and 3, respectively (Cholongitas et al., 2014and Sulkowski et al. in 2014). It was also in agreement with a study done by Welzel et al. [20], where SVR12 was achieved by 91% of patients. In contrast to Fontaine et al. in 2015, cirrhotic patients receiving SOF + DCV + RBF and sofosbuvir and simprevir achieved a 100% SVR thus was considered a good therapeutic option. This could be due to the fact that those patients not only were given additional regimen but also were treatment-experienced patients unlike any of the naïve patients in our study. Furthermore, most of the patients in this study are genotype 4 patients with lesser extent genotype 1, 2, and 3. Finally, it is concluded that the best treatment regimen among the 4 groups is that of group D having the highest SVR12 of 96%, highest tolerability, and least side effects [21–25, 2, 26, 27].

## Recommendations

To generalize results, greater sample size, more female patients, and treatment of the patients who relapsed with other new DAA treatment regimens should be taken into consideration.

## References

[1] Amer FA (2018). Large-scale hepatitis C combating campaigns in Egypt and Georgia; past, current and future challenges. J Infection in Developing Countries.

[2] Reker C, Islam KM (2014). Risk factors associated with high prevalence rates of hepatitis C infection in Egypt. Int J Infect Dis: IJID: Official Pub Int Soc Infects Dis.

[3] Dore G, Ward J, Thursz M (2014). Hepatitis C disease burden and strategies to manage the burden. J Viral Hepatitis.

[4] AU J, Pockros P (2014) Novel therapeutic approaches for hepatitis C. Clinical Pharmacol Ther 95(1):78–88 10.1038/clpt.2013.206 24126682

[5] Kouyoumjian SP, Chemaitelly H, Abu-Raddad LJ (2018). Characterizing hepatitis C virus epidemiology in Egypt: systematic reviews, meta-analyses, and meta-regressions. Sci Rep.

[6] Tomer Y, Blackard JT, Akeno N (2007). Interferon alpha treatment and thyroid dysfunction. Endocrinol Metab Clin North Am.

[7] Sonnenblick M, Rosin A (1991) Cardiotoxicity of interferon. A review of 44 cases. Chest 99(3):557–561. 10.1378/chest.99.3.557 10.1378/chest.99.3.557 1704826

[8] Chehadeh W, Abdella N (2009). Risk factors for the development of diabetes mellitus in chronic hepatitis C virus genotype 4 infection. J Gastroenterol Hepatol.

[9] Bruno S, Shiffman M (2010). Efficacy and safety of peg interferon alpha-2a (40KD) plus ribavirin in hepatitis C patients with advance fibrosis and cirrhosis. Hepatology.

[10] Gramenzi A, Andreone P (2001). Impact of interferon therapy on the natural history of hepatitis C virus related cirrhosis. Gut.

[11] Swain M, Lai M (2010). A sustained virologic response is durable in patients with chronic hepatitis C treated with peg interferon alpha-2a and ribavirin. Gastroenterology.

[12] Kumar D (2003). Effectiveness of IFNα-2b/ribavirin combination therapy for chronic hepatitis C in a clinic setting. Med J Aust.

[13] Ward RP, Kugelmas M (2005). Using pegylated interferon and ribavirin to treat patients with chronic hepatitis C. Am Fam Physician.

[14] Kemp W, Roberts S (2011) Pegylated interferon and ribavirin for the treatment of chronic hepatitis C. Clin Med Insights: Ther 3:CMT.S4015. 10.4137/CMT.S4015

[15] Elmashad M, Elsherif A (2017). Comparative study between peg IFN based regimen (peg-IFN/sofosbuvir/ribavirin) and peg IFN free regimens (sofosbuvir/simprevir)-(sofosbuvir/ribavirin) in Egyptian patients with chronic HCV infection. Eur J Pharm Med Res.

[16] Dolatimehr F, Haron-Sari et al (2017) Combination of sofosbuvir, pegylated interferon and ribavirin for treatment of hepatitis C virus genotype 1 infection: a systemic review and metaanalysis. DARU J Pharm Sci 25:11 10.1186/s40199-017-0177-x PMC5397824 28427463

[17] Kowdley KV, Lawitz E, Crespo I, Hassanein T, Davis MN, DeMicco M, Bernstein DE, Afdhal N, Vierling JM, Gordon SC, Anderson JK, Hyland RH, Dvory-Sobol H, An D, Hindes RG, Albanis E, Symonds WT, Berrey MM, Nelson DR, Jacobson IM (2013). Sofosbuvir with pegylated interferon alfa-2a and ribavirin for treatment-naive patients with hepatitis C genotype-1 infection (ATOMIC): an open-label, randomised, multicentre phase 2 trial. Lancet (London, England).

[18] Doss W, Shiha G, Hassany M, Soliman R, Fouad R, Khairy M, Samir W, Hammad R, Kersey K, Jiang D, Doehle B, Knox SJ, Massetto B, McHutchison JG, Esmat G (2015). Sofosbuvir plus ribavirin for treating Egyptian patients with hepatitis C genotype 4. J Hepatol.

[19] Aboud A (2017). Study of the efficacy of triple therapy of sofosbuvir; pegylated IFN alpha 2a and ribavirin in treatment of chronic hepatitis patients genotype 4 with high fibrosis. Open J Gastroenterol.

[20] Welzel TM, Peterson J, Et Al. Daclatasvir plus sofosbuvir, with or without ribavirin achieved high SVR rates in patients with HCV infection and advanced liver disease in a real-world cohort. GUT 2016. 10.1136/gutjnl-2016-312444 PMC5099229 27605539

[21] Evangelos C (2014). Papatheodoridis et al, Sofosbuvir: a novel oral agent for chronic hepatitis C. Ann Gastroenterol.

[22] EASL recommendations on treatment of hepatitis C 2016. J Hepatol Xxx:5 10.1016/j.jhep.2016.09.001 27667367

[23] Fontaine H, Hezode C et al (2015) Efficacy of the oral sofosbuvir-based combination in HCV genotype 4 mono-infected patients for the french observational cohort ANRS Co22 HEPATHER. Abstract LP 28 Presented At The 50th Annual Meeting Of The European Association For The Study Of The Liver; Vienna

[24] Martinot-Peignoux M, Stern C (2010). Twelve weeks post-treatment follow-up is as relevant as 24 weeks to determine the sustained virological response in patients with hepatitis C virus receiving pegylated interferon and ribavirin. Hepatology.

[25] Omran D, Alboraie M, Zayed RA, Wifi M-N, Naguib M, Eltabbakh M, Abdellah M, Sherief AF, Maklad S, Eldemellawy HH, Saad OK, Khamiss DM, El Kassas M (2018). Towards hepatitis C virus elimination: Egyptian experience, achievements and limitations. World J Gastroenterol.

[26] Sulkowski MS (2014). Daclatasvir plus sofosbuvir for previously treated or untreated chronic HCV infection. NEJM.

[27] Yoshida E, Sulkowski M (2015). Concordance of sustained virological response 4, 12, and 24 weeks post-treatment with sofosbuvir containing regimens for hepatitis C virus. J Hepatol.

